# Student comprehension of biochemistry in a flipped classroom format

**DOI:** 10.1186/s40561-024-00356-z

**Published:** 2024-12-04

**Authors:** Edward N. Harris, Evan A. Schroder, Teryn J. Berks

**Affiliations:** Department of Biochemistry, University of Nebraska – Lincoln, 1901 Vine St., Beadle N133, Lincoln, NE 68588 USA

**Keywords:** Flipped, Inverted, Classroom, Biochemistry, Undergraduate

## Abstract

Concept-heavy courses such as Biochemistry in life and physical science curricula are challenging for many college-aged students. It is easy for students to disengage in a lecture and not learn the subject matter while in class. To improve student learning *and* participation, we employed a flipped format for the first half of the course and compared learning outcomes and attitudes with the traditional lecture in the second half of the course. The experimental course was an upper-level biochemistry course taken primarily by juniors (49%), seniors (40%), and some graduate students (10%) at the college level. Our results indicate that lower-performing students academically benefited from the flipped format (twofold lower failure rate as compared to previous years), whereas, there was no change with higher performing students when comparing student grades from previous courses. Academic assessments were derived from exams, quizzes, in-class activity/participation, and out-of-class homework. Participation and overall satisfaction with the course was significantly much higher in the flipped format (88% of students) than in the traditional lecture (< 10%) for all students. In-class activities in both faculty preparation and execution were crucial to the success of the flipped format. Our conclusions are that the flipped pedagogy is better for the reinforcement of difficult concepts for struggling students (95% students in agreement) and merits higher participation than the traditional lecture. Two-way communication between instructor and students is encouraged and enhanced with in-class activities and case studies. Due to the success of this project and in agreement with ~ 75% of the students, the entire course will be flipped going forward.

**Supplementary Information:**

The online version contains supplementary material available at 10.1186/s40561-024-00356-z.

## Introduction

The goal for educators in Science, Technology, Engineering and Mathematics (STEM) disciplines is to foster understanding of difficult concepts that are inherent in these disciplines. Some common STEM courses in the undergraduate level are pre-requisites for upper-level courses in the major and for professional schools. As a result, students from diverse backgrounds and majors are enrolled in these courses, particularly physics, general chemistry, general biochemistry, with a high demand for students to comprehend enough material to be proficient at some arbitrary level (Toth & Montagna, 2002). Continual student engagement in highly dense informational STEM courses is challenging in the traditional lecture format (Patel et al., 2019). To address this, educators have flipped their classrooms in which the lectures are provided online before class and class time is spent on the demonstration of application problems, case studies, question/answer periods, and other activities that engage the students (Luker et al., 2015; Shi et al., 2019; Yoder et al., 2019; Zappe et al., 2009). The addition of added activities in the classroom other than just a lecture promotes student centeredness and increases conceptualization of STEM principles (Struyf et al., 2019). Flipping the classroom is not a new technique (Mazur, 1999), but has become a more popular pedagogy in the past 15 years. In fact, there are instructional guides for implementing coursework in a flipped classroom format (Poulain et al., 2023). The outcomes of flipping biochemistry courses at the university and medical school level have resulted in improved student satisfaction and engagement in the course (Fakhoury et al., 2021; Låg & Sæle, 2019), motivate and promote better learning (Alonso-Chamorro, 2019), and even increase student understanding of the primary research literature (He & Masuda, 2015). Variants of flipping have also included individual versus cooperative flipped formats (Jafarkhani & Jamebozorg, 2020), integration with team-based learning (Ji et al., 2023; Zaman et al., 2022), and massive open online courses (MOOCs) (Zhang et al., 2020).

The flipped format does not solve all learning problems with students. Some students may not enjoy active learning or collaborate well with others (Ding & Zhu, 2021; Stöhr et al., 2020). Additionally, the instructor may not invest enough time and effort in the preparation of in-class instructional material (Long et al., 2017). There is some anecdotal evidence that if a student is enrolled in multiple flipped courses, the student may be overwhelmed with the volume of recorded material to review every day, affecting attitudes with the courses (Smith, 2013). The change in format, along with perceived extra work outside of the classroom to watch lectures, may produce feelings of anxiety, resistance to change, and adoption problems in students (Akçayır & Akçayır, 2018; Chen et al., 2014). To avoid or overcome obstacles, instructors should ensure students are well prepared for in-class activities, be organized in their instructional methodology, design learning materials and activities based on student feed-back, develop cooperation and sharing skills, and provide support within the class, especially for those who are struggling to understand the subject matter (Long et al., 2017; Poulain et al., 2023). Thus, the most important task for the instructor is the refinement of course pedagogy as a continual process to enhance student learning and satisfaction with the course.

### Statement of the problems

Anecdotal statements about traditional lecture indicate that many students are accustomed to this methodology for teaching. However, in our experience there are a few persistent problems associated with the traditional lecture. First, in courses with a high amount of information given in each class period, it is easy for students to be overwhelmed (Nielsen, 2023). Overwhelmed students will either “zone out” or get mentally “lost” and rarely re-engage the class. Poorly engaged students lack the learning required to achieve the course objectives (Li, 2022). This may lead to a lost opportunity to learn the material. Second, the classroom time is 75 min long, which is beyond the attention span of many individuals concerning the amount and level of material that is required to learn. For some subjects, the twice per week class that is about 1.5 h long per session/class time is optimal (Reardon et al., 2008), but may be too long for some STEM subjects (Palocaren et al., 2016). Third, students come from a variety of disciplines and may not be as well prepared as their peers, even though they all take the same prerequisite courses such as general chemistry, algebra, calculus, and physics. Due to this variability, learning instruments have been developed to address this problem. For example, the Organic and Biochemistry Readiness Instrument (ORBI) reinforces critical knowledge gaps in inorganic chemistry to prepare students for organic and biochemistry courses (Torres et al., 2024). Unfortunately, ORBI nor any similar instrument was not applied here. This course (Biochemistry I) is a requirement for many health professions (medical, dental, pharmacology, etc.) and biochemistry majors. Therefore, students majoring in humanities, for example, are still held to the same academic performance bar as biochemistry majors. To address our observed classroom problems (poor engagement, long class, variable readiness), we asked the following question, “Does changing format and delivery of the material rather than the content increase student comprehension or performance in the course objectives of Biochemistry? This report on the results of our course should reinforce the notion that flipping a biochemistry course facilitates student learning through higher classroom engagement and repeated exposure to fundamental concepts.

## Methodology

### Demographics of the biochemistry course

The flipped classroom was implemented in a large undergraduate upper-division biochemistry course at a large research-intensive (R1) university in the Midwest region of the United States. The class had 105 students and consisted of 60% male and 40% female. About half of the students were juniors in their 3rd year of studies and 40% were seniors. The remaining 10% consisted of graduate students taking the course as a refresher as part of the PhD academic plan and there was one freshman (Table 1).

**Table 1:** Demographics

Population	% of students
Male	60
Female	40
Freshman	0.9
Sophomore	0
Junior	49
Senior	40
Graduate	10
Biochem Major	30
Non major	70

Thirty percent of the students were biochemistry majors, and a majority of nonmajors had declared their major course of study in Biological Sciences. The remaining minority consisted of students from about a dozen other disciplines (Table 2). All the undergraduates had the same pre-requisite requirements whether they were biochemistry majors or non-majors. Pre-tests were not administered to assess baseline knowledge.

**Table 2:** Non-major disciplines

Non-majors	% of students
Biol. Sci	41
Education, human Sciences	1.3
Engineering	8
Microbiology	13
Nutrition	13
Anthropology	1.3
Finance	1.3
Psychology	7
Math	1.3
Animal Sci	9.2
Plant Biology	1.3
Chemistry	1.3

### Ethics approval and consent

The Internal Review Board of the university approved protocol IRB #20230622913EX that included all activities and curricula in this course. Students filled out a consent form on the first day of class without the instructor present and administrative staff collected the consent forms which were not available to the instructor until after final grades were submitted.

### Traditional lecture format of the Biochemistry course (weeks 8–15)

**Fig. 1:**
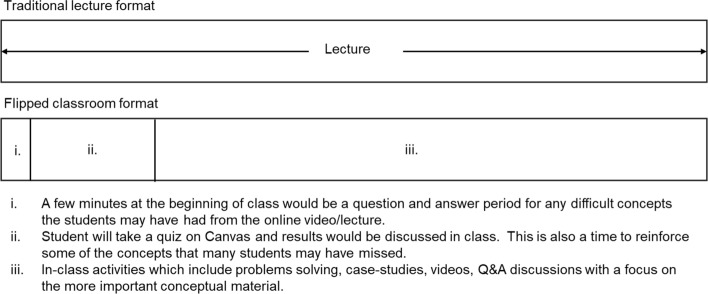
Class time organization. The utilization of class time in both the traditional lecture and flipped formats. The lecture is on continuous activity, whereas, the flipped course contains at least 3 different activities

In prior semesters before flipping the course, the scheduled lecture times were every Tuesday and Thursday of the week with classes that were 75 min long throughout a 15-week semester (Table 3**, **Fig. 1). This is equivalent to a standard three credit-hour course. Recitations are held every Friday afternoon for an hour with the instructor. Recitations do not require attendance and are primarily for those who want extra help in the course. In the traditional lecture format, weekly homework assignments were distributed on each Thursday and were due on the following Tuesday. The homework assignment covered the material presented in the week of when it was distributed. Two teaching assistants were available to grade homework, exams, and meet with students for up to 4 h/week throughout the semester. Lectures were recorded in class and posted to Canvas after class for those who may have missed class and for those who wanted to review the lecture as part of their study habits. iClicker technology was used in lectures to informally assess student comprehension.

**Table 3:** Class organization

	Monday	Tuesday	Wednesday	Thursday	Friday
Traditional		75 min Lecture HW due from previous week		75 min Lecture HW passed out and due next Tuesday	Recitation by Instructor
Flipped	Students watch asynchronous online lecture	1. Short quiz on lecture principles 2. Short lecture on difficult concepts 3. Classwork and activities	Students watch asynchronous online lecture	1. Short quiz on lecture principles 2. Short lecture on difficult concepts 3. Classwork and activities	Recitation by Instructor

### Implementation of the flipped class format (weeks 1–7)

#### Lectures

All lectures were pre-recorded and posted in Canvas, which is the Learning Management System available to all students at the university. Lectures were presented with PowerPoint slides with audio voice-over of the instructor. Each lecture was 30–60 min long depending on the amount of material that was presented and amenable for this type of presentation without student input. Lectures were made available at least 2 days prior to class times which means that all students had the opportunity to review the lectures on Mondays and Wednesdays prior to meeting on Tuesdays and Thursdays.

#### In-class activities

The first activity at the start of class is to take a comprehension quiz based on material that was present in the prior online pre-recorded lecture. The quiz was administered via Canvas and student could easily do this on their phones and laptops. The quiz questions were based on basic comprehension topics that did not involve much memorization or detail. The goal of the quizzes was to ensure that students listened to the recorded lecture and prepared themselves for the discussion and activities in the classroom. The second activity was to ask students about what were the “muddiest” points of the recorded lecture. This was to identify the most difficult concepts that students had trouble understanding. This was asked by a show of hands by the instructor or the instructor putting up a list of topics and students selecting by ranking which topics needed to be discussed the most. This part of the class lasted only about 10–15 min. The remaining 40–45 min of class was spent on the third activity which consisted of doing problems that were similar to the homework problems in the traditional lecture classes. The instruction would often incorporate scaffolding approaches (Belland, 2017; Siller et al., 2023). Normally, the instructor would do one as a demonstration of how to logically approach the problem and derive an answer. The second problem would then be given to the students in which they could work individually or in groups, depending on the difficulty or complexity of the problem. Sometimes the instructor would solve part of the problem and students would be asked to finish. There would often be discussions in class about why and what rationale is inherent in the problem or problem set. Students would each get a worksheet in which they could take notes and self-application of these in-class activities.

#### iClicker

The rationale for the use of iClicker over other response systems is that iClicker is integrated with Canvas and iClicker is widely used at the university in lower-division courses. There were two goals for using iClicker in this course. The first goal was to measure attendance, which was recorded throughout class time. The second goal was to get responses from students on survey questions that were integrated with the in-class activities, including muddiest point discussions and answer surveys for problems that were presented in class.

#### Grading

Four exams were given throughout the course with the first two exam coinciding with the flipped format and the last two exams based in traditional lecture format. These four exams consisted of 68% of the course grade. Other components of the grade include participation via iClicker (9.5%), in-class quizzes (8.5%), and homework during the traditional lecture series (14%). Final grades were distributed on a 10% scale (90–100 = A, 80–89 = B, 70–79 = C, etc.).

#### Statistics

This course was administered in the Fall semester (Aug–Dec) of 2023. All single group comparisons were calculated using the Student’s T test. For calculations in which this was not appropriate, comparisons were calculated using the Mann–Whitney algorithm. Multi-group comparisons were performed by one-way ANOVA statistical analyses. All statistical calculations were performed with SigmaPlot version 11.2 software.

## Results

### Online lecture viewership

**Fig. 2:**
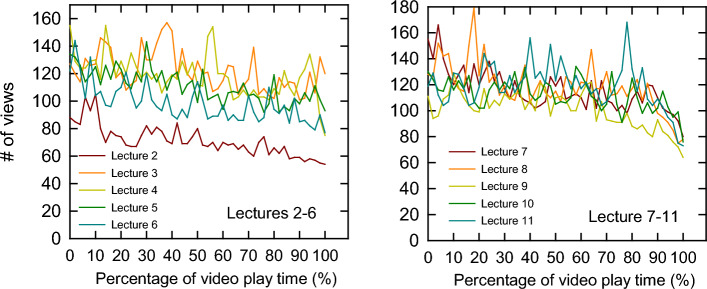
Online recorded lecture viewership. YuJa software embedded in Canvas LMS recorded views every 5 seconds during each lecture and the length of time vs views was normalized to 100%, regardless of play time. The first and last minute of each lecture was not included in the data due to false starts and end-times that were silent due to how the lecture was recorded by the instructor. Peaks within each line plot represent repeated views by students

Canvas is the learning management systems (LMS) used by all courses at the university so students are very familiar with this software. All videos were pre-recorded using a 3rd-party vendor, Yuja, that is integrated with Canvas and videos were posted at least two days prior to meeting in class. Yuja software is able to gather video analytics and, from the eleven flipped courses that were posted, viewership remained consistent throughout the entire length of the video. Most lecture videos had a small drop-off of about 12% in the last few minutes, but overall, viewership is robust (Fig. 2). There are a few peaks in the data suggesting that students were rewatching specific segments of the video. This was confirmed in several discussions with students as some of them related that if the material was in any way confusing or difficult to understand, they would re-watch certain segments.

### Quiz and participation performance

**Fig. 3:**
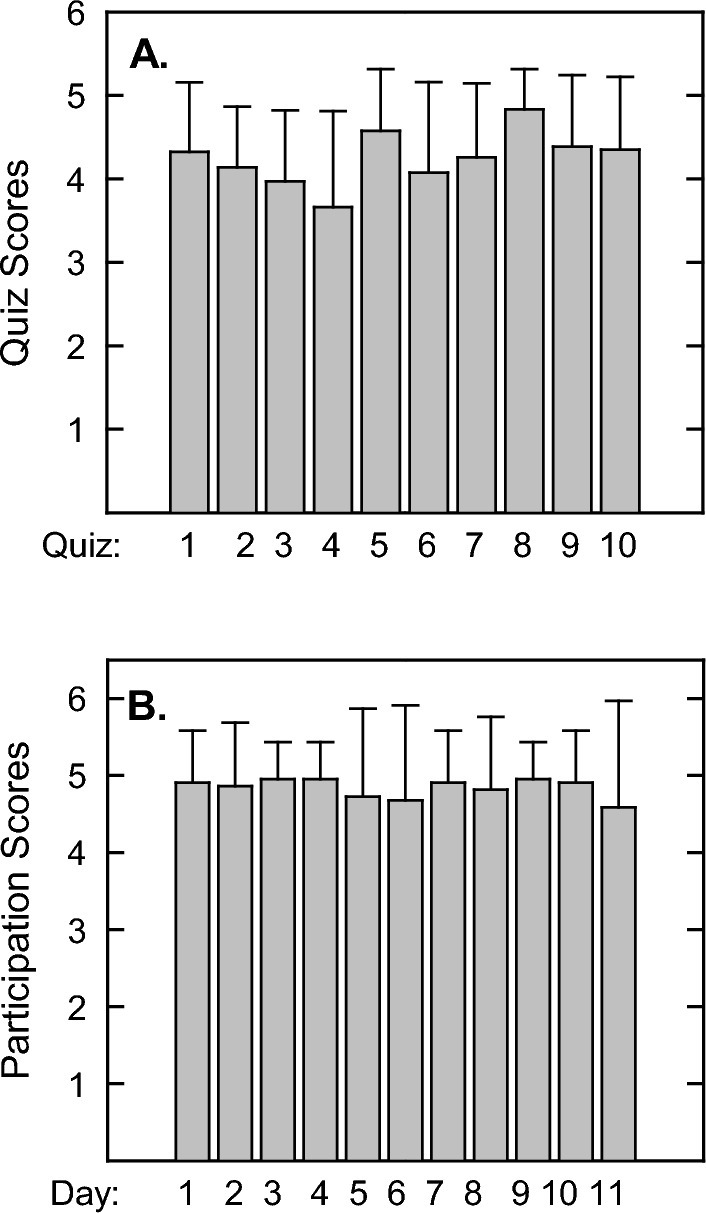
Quiz and Participation scores during the flipped format. (**A**) In-class quizzes (5 pts each) were administered at the beginning of each class and recorded in Canvas LMS, n = 105 using one-way ANOVA with significance of p ≤ 0.05 and Q>2.55. (**B**) Participation during in-class activities were recorded by iClicker with at least one response required for the full 5 points, n = 105

For each flipped class period, one of the first activities of the day was a short 5-min quiz administered on Canvas. The primary purpose of the quizzes were to motivate student to come prepared for class by watching the online lecture. Students were given 5 questions with multiple choice answers that covered the basic concepts of the online lecture made available at least 2 days prior to class. The quiz was timed, and the students had one chance to make their answer selection. Immediately after the quiz, the instructor would review each question with the students to clarify any misunderstandings. Results of the quizzes indicate that students averaged 4.25/5 points per quiz and the standard deviation revealed that the vast majority of students did not score lower than a score of 3/5. However, there were a few outliers in which students scored 0–2 points per quiz. Using a one-way ANOVA test using all pairwise multiple comparison procedures or Dunn’s Method in which the cut-off for significance is p < 0.05 and a Q value of greater than 2.55, all the scores of the following quiz dates were significantly different from each other except for the following comparison days: 5 versus 8, 5 versus 7, 2 versus 9, 1 versus 3 and 4 versus 6 (Fig. 3A). It is notable that there is a downward trend in the first four days that rebounds again. This may have resulted from the rigor of the quizzes and that students were reminded to avoid complacency after the 4th quiz since these quizzes had a significant impact on final grades.

Participation was measured with the use of iClicker. In the presentation of individual and group problem solving during class activities, the instructor would post a question with multiple choice answers on the overhead screen in which the students had to select a correct answer. Most students used a cell phone or laptop using iClicker software; however, there were a few students who had the iClicker device and some of these were problematic. To accommodate these few students using outdated iClicker hardware, the instructor had a printed sheet of problem sets and these were turned in at the end of class time and counted toward their participation score. Participation was either all or nothing with the threshold of answering at least one question by iClicker. Typically, there were at least 3 questions for each flipped class day. Using the same ANOVA test to compare multiple groups, a comparison of the medians were significantly different (*p* = 0.014), however, the Q values were all too low, so overall, there was no real statistical significant differences between participation levels for each of the flipped class days (Fig. 3B).

### Exam performance

**Fig. 4:**
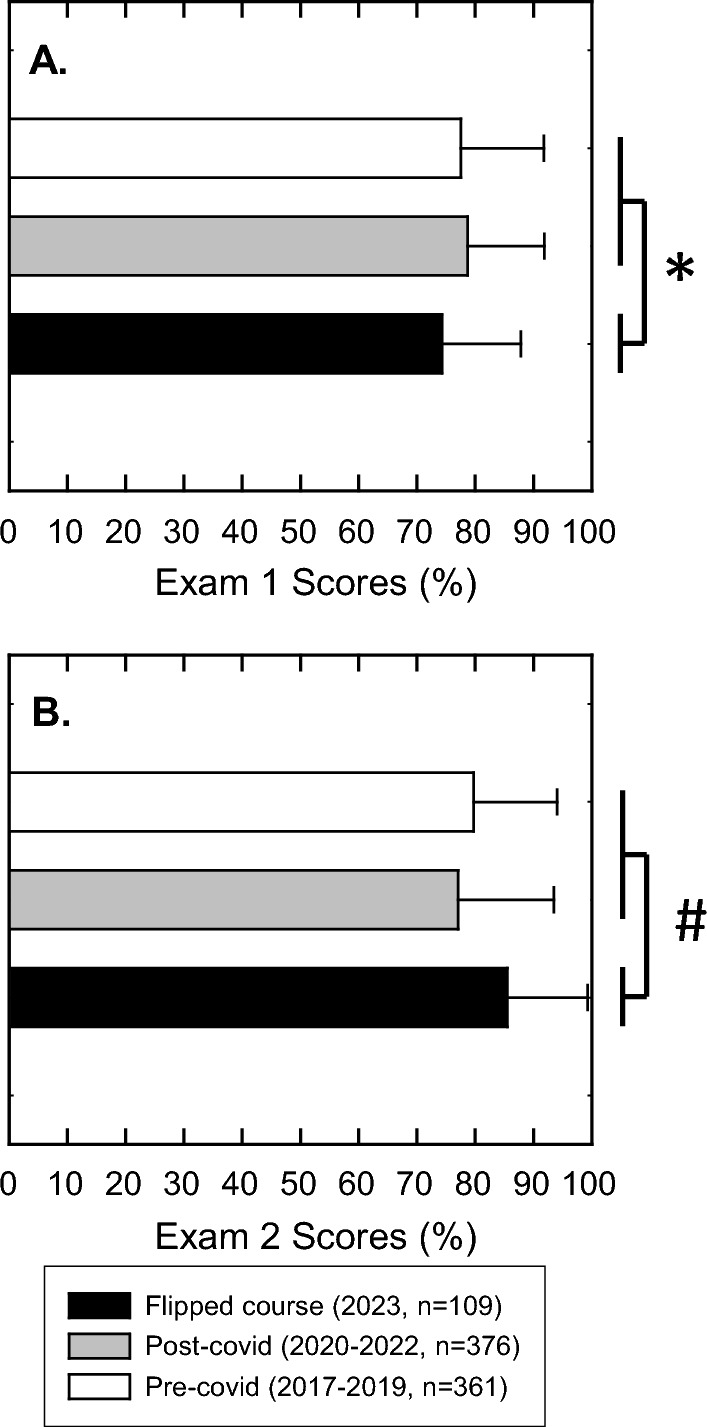
Exam scores. Exam scores from 3 pre-Covid and 3 post-Covid courses in the traditional lecture were compared with the flipped course (**A**) Exam 1 and (**B**) Exam 2. Statistical analysis by Student’s T test followed by the Mann–Whitney U test revealed that the both pre- and post-Covid exams were not significantly different in Graph A (p = 0.148) and in Graph B (p = 0.07). However, the flipped course exam 1 and exam 2 were significantly different from the traditional lectures *p = 0.002 and #p < 0.001

The course had 4 exams throughout the semester that were graded with equal weight values. The first two exams covered the flipped course and the last 2 exams covered the traditional lecture. Our inquiry was to find out if student performance was improved with the flipped classes. To do this, we compared the same content that was taught in the traditional lecture in pre-covid (2017–2019) versus post-covid (2020–2022) versus the flipped content (2023). Comparing the traditional lecture pre- and post-Covid for exam 1, the difference is insignificant (*p* = 0.148) using the Student’s T test. However, the difference between the post-Covid exam 1 and the flipped class exam 1 was different (*p* = 0.002) in that the student scored lower in the flipped class on average by several points (Fig. 4A). Likewise, for exam 2, the pre- and post-Covid exams were not significantly different (*p* = 0.07); however, the exam outcome for the flipped course exam 2 was higher (*p* < 0.001) by an average of 9 points (Fig. 4B). The significantly higher outcome for the exam was correlated to higher satisfaction with the course organization and presentation of material. From our perspective, students were becoming more accustomed to the format of the course and student engagement remained high.

### Mid-course survey

**Fig. 5:**
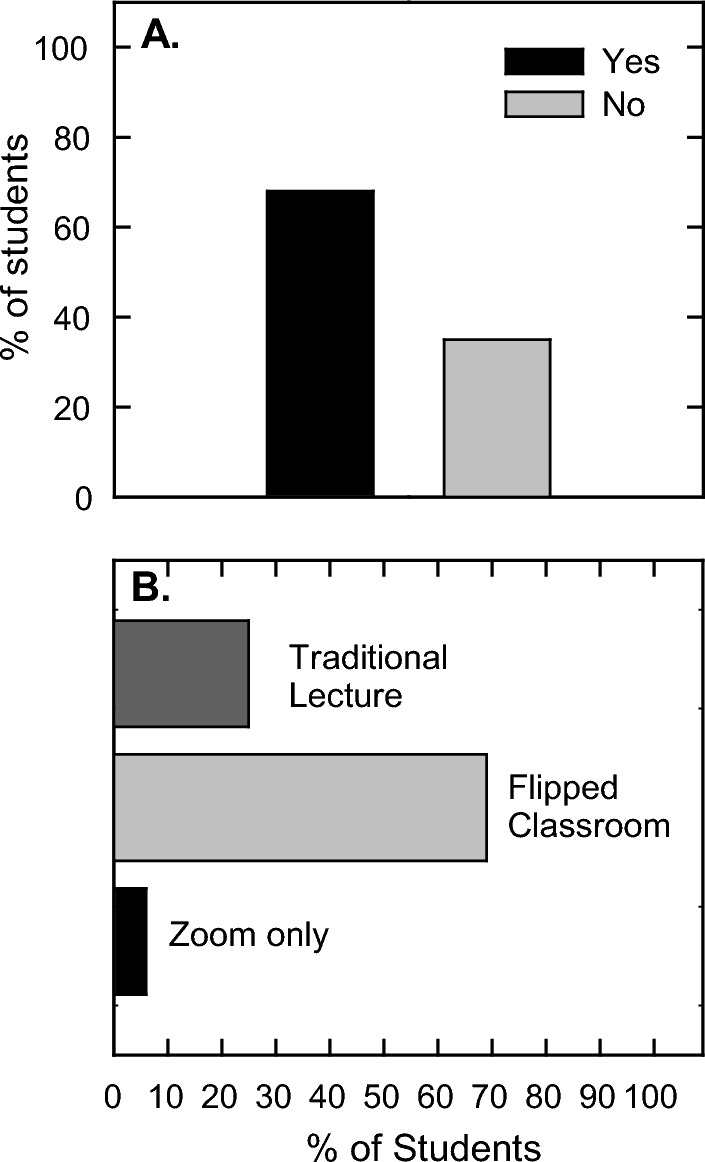
Brief mid-term survey. The questions for (**A**) Is this course structure working for you? and (**B**) What class format works best for you? were informally surveyed in class using iClicker. n = 102

During the fourth week of the course during lecture 8, which was in between exams 1 and 2, an informal survey was presented during class and recorded by iClicker. The motivation for this survey was to ensure that the instructor was deploying the course with intended results due to the outcome of the first exam. The first questions was “Is this course structure working for you?” (Fig. 5A). The result was that 2 out of 3 students (68%) confirmed that the outlay of the course and their ability to learn the material within this structural organization was satisfactory. The second question was “What class format works best for you?” and this focused on what students liked as far as their comfort level with course organization (Fig. 5B). Approximately the same results were given in that (69%) indicated that the flipped format works best for them. For those who disagreed with this format, 25% confirmed that they enjoyed the traditional lecture and 6% were more comfortable with Zoom only courses. It should be noted that the student’s perception of traditional lecture is from previous courses as traditional lecture had not yet been practiced in this course.

### End-of-semester survey

**Fig. 6:**
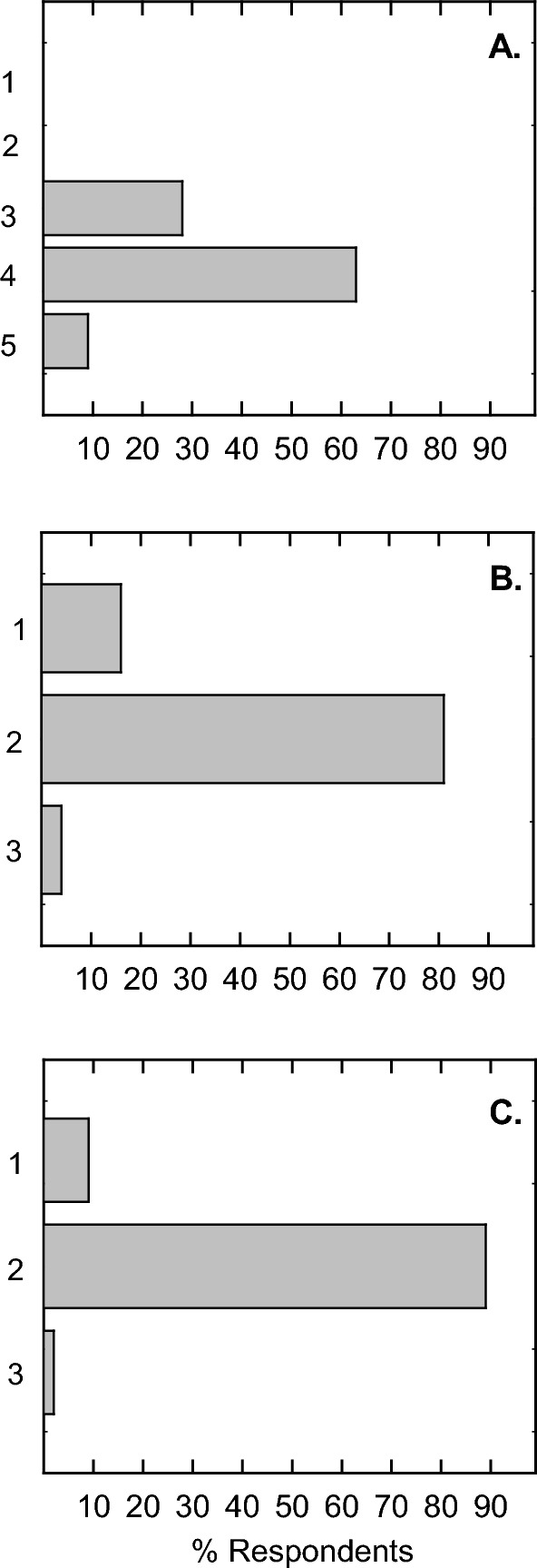
End-of-semester survey on difficulty, content, and format. (**A**) Students were survey with the question “How difficult is this course?” with 1 = easy, 2 = moderately easy, 3 = slightly challenging, 4 = difficult, 5 = very difficult. (**B**) Students were surveyed with the question “Was the content of this material 1) more difficult than it should be, 2) About right for an upper division biochemistry course, 3) Generally easy”. (**C**) Students were surveyed with the question “What format do you prefer learning this course material 1) Traditional Lecture, 2) Flipped, 3) Zoom only”, n = 57

During the last week of classes, the students were given an anonymous survey to assess their experience with both the flipped and traditional formats of the same course. Of the 105 students that were enrolled in the course, 57 students responded. The motivation for an anonymous survey was to include an honest write-in answer to the question “What advice would you give the instructor to make this class better?” at the end of the survey. There was no extra credit or grade for this survey and students filled it out on their own motivation to improve the course. Anonymity promoted critically honest answers and their written responses are found in Fig. S1. A majority of the students enrolled in the course found it difficult to very difficult with 28% responding that it was moderately difficult (Fig. 6A). Concerning the content, most students (81%) found that the difficulty was on par for an upper-level division biochemistry course with 16% and 4% citing that it was either too difficult or too easy, respectively (Fig. 6B). After experiencing both flipped and traditional formats of the course, 89% preferred the flipped format, 9% preferred traditional lecture style, and 2% preferred Zoom-delivered course only which is online lecture and no class attendance required (Fig. 6C).

**Fig. 7:**
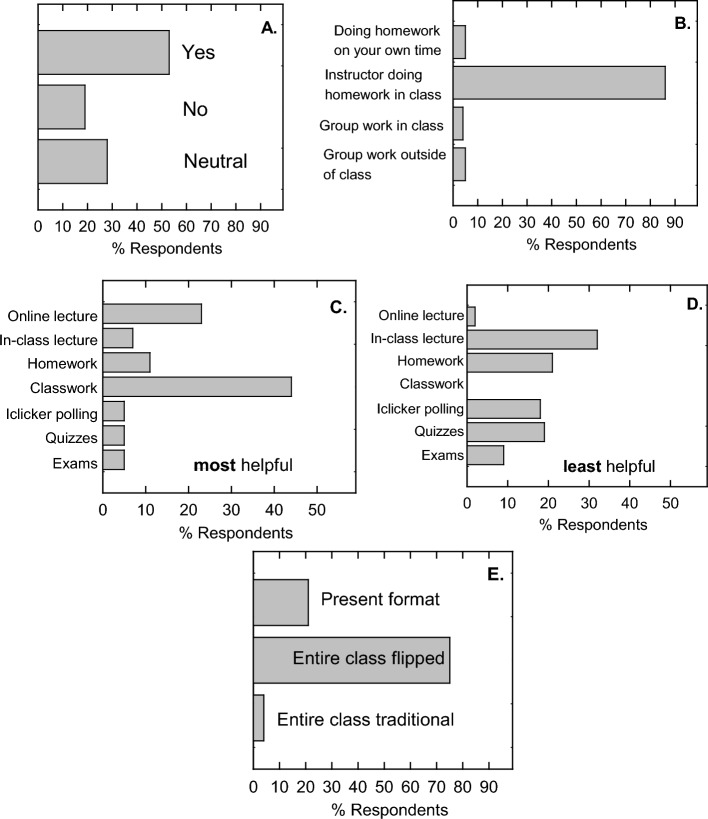
End-of-semester survey on course activities and structure. (**A**) Students were surveyed with the question “During the flipped portion of the course, were the in-class quizzes helpful for understanding the material?” (**B**) Students were surveyed with the question “With regards to learning the concepts of biochemistry, which activity do you prefer?” (**C**) Students were surveyed with the question “What was the most helpful activity of the course for learning biochemistry?” (**D**) Students were surveyed with the question “What was the most helpful activity of the course for learning biochemistry?” and (**E**) Students were surveyed with the question “If you had to take the course again, what format would you prefer?” n = 57

Activities were an important part of the flipped classroom portions of the course. To determine if the quizzes were actually helpful for the students, about half of those surveyed (53%) said that they were helpful in learning the material (Fig. 7A). The other 47% responded in either the no or neutral category. Concerning learning the concepts of the course, 86% of those surveyed preferred that the instructor work out the problems with students in class (Fig. 7B). This consumed most of the time of in-class activity. Group work in-class or out-of-class as well as traditional homework were all less than 10% preferred, strongly suggesting that new concepts are better understood when demonstrated in the classroom setting in an interactive format. Their next question focused on what was most and least helpful in the course. The clear stand-out answer for the most helpful aspect of the course was the instructor doing homework problems and interacting with the class followed by the online lecture (Fig. 7C). The least helpful aspect of the course was the in-class lecture during the traditional lecture part of the class (Fig. 7D). It was also interesting to note that no one voted for classwork as the least helpful part of the course. The last surveyed question was “If you had to take the course again, what format would you prefer?” The majority (75%) would have liked to see it flipped, while 21% preferred the present course structure of the first half flipped and the second half traditional. Only 4% would have preferred the traditional format (Fig. 7E).

## Discussion

Students entering this course had no familiarity with the flipped course format. All of them came from traditional lecture pre-requisite courses that used a variety of teaching methods such as backward design, think-pair-share, and other activities that supplemented traditional lecture methodology. Students were a bit slow to catch on to the flipped format and that is evident in lecture 2 (Fig. 2) which had the lowest viewership of all the recorded lectures. This lecture is also the first “flipped” lecture in the course. In the literature, comparative studies often split biochemistry classes into a flipped class or a blended-learning class with a traditional control (Halasa et al., 2020; Oliván-Blázquez et al., 2023). However, this course combined the flipped with traditional teaching methods which is not unprecedented (Swithenbank & DeNucci, 2014). The reason why we selected this pedagogy is that some of the subject matter tended to be more amenable for better classroom activities while other subjects were more difficult for making meaningful activities. Course material that was dependent on mathematical equations and problem solving tend to be more intuitive for in-class activities, in contrast to material that tends to focus more on pathway analysis. The level of difficulty remained about the same according to the majority of students as this course did not start out easier than it ended. Going forward, we plan to flip the entire class and diversify the types of activities that we are planning for class.

**Fig. 8:**
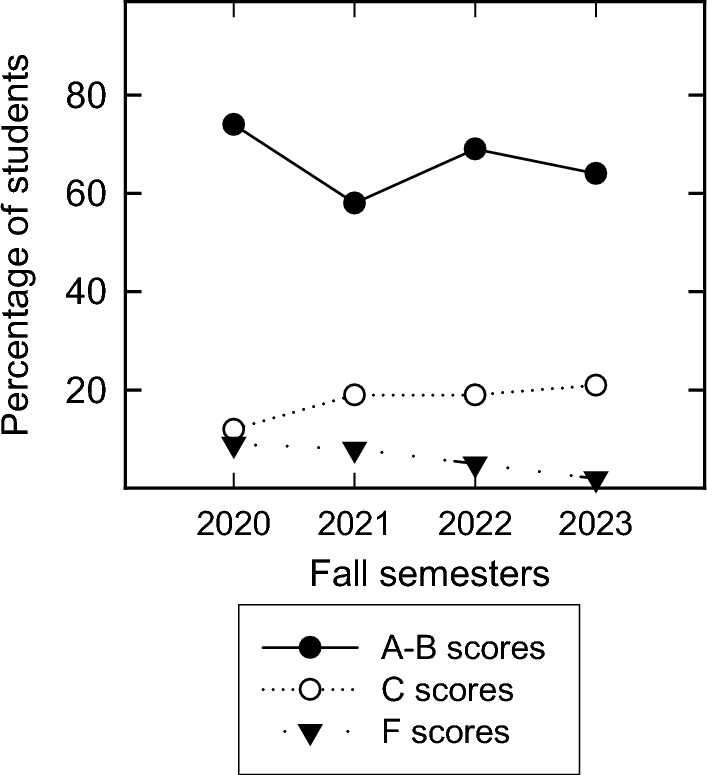
Final grades for this course. Percentage of passing and failing grades of students in the course (2020-2023). It should be noted that years 2020-2022 were traditional lecture and year 2023 was the flipped course.

Was there an improvement in student overall grades? By the second exam, the answer would be yes. However, taking in all the scores accumulated across all 4 exams (2 flipped, and 2 traditional), there was no significant difference in the final grades as compared to previous traditionally taught classes, with the exception in one area. The course had a twofold lower number of failing students at 2% of the class. In contrast, in the prior 3 years, the failure rate was 9%, 8% and 5% for the years 2020, 2021, and 2022 (Fig. 8). For these 3 years, the failure rate has been trending downward anyway, so it is impossible to know if the 2% failure rate follows that trend or if the flipped pedagogy actually helped those students who were having major academic problems. In this course, 2% represented 2 students who stopped showing up to class during the traditional lecture and never dropped the course. Therefore, it is probable that the students who were attending class throughout the semester benefited. The cohort of students who earned C grades increased to 21% from 19% in 2022 and 2021 and 12% in 2020. Students earning A and B grades were always high (64% in 2023, 69% in 2022, 54% in 2021, and 74% in 2020) suggesting that the current flipped/traditional pedagogy helped the students who were struggling but had no real impact on high achievers earning A and B scores. Published results from the flipped pedagogy have varied. There are some who argue that even though students are more engaged in the flipped format, there is no actual improvement in student scores (Gough et al., 2017; Smith, 2015), which is seen more in the K-12 classes. This may be attributed to many other factors concerning young children as they live at home with many other things going on. With college students, flipping the class has the benefit of increasing the learning capabilities of those who normally struggle (Fulton, 2012; Johnson, 2013; Zawilinski et al., 2020). This is evident in more recent meta-analyses comparing many of these studies to assess college student learning outcomes. The flipped pedagogy is directly linked with increased student learning across a wide variety of STEM courses at the college level, but there is a major caveat to this. Consistently, when instructors do not plan the classroom activities very well, there is no guarantee that the flipped pedagogy will improve student learning (Cheng et al., 2019; Shi et al., 2019). It is easy to fall back on re-lecturing which commonly occurs when there is a lack of activity preparation or poor online lecture quality. From the instructors’ perspective, the amount of work it takes to prepare the course doubles since good quality video must be recorded and the material for class also needs to be prepared (Taylor, 2015). From our experience, developing good worthwhile classroom activities that enhance what the student previously viewed online is the most difficult and challenging aspect of developing and executing a flipped class. One of the major impediments that we encountered was that the classroom had fixed, theater-style seating so group work and dividing the class into subsections was difficult to achieve. All the group work that we used in this course was very transient (think-pair-share) and only utilized 2–3 people in groups due to physical limitations of the physical classroom.

The effects of activity and participation in the development for learning and memory retention are well-known for decades (Brown & Oxman, 1978; Gathercole et al., 2008; Gathercole et al., 2006). At the college level, high performing students who do well often can conceptualize and understand new information. Students that cannot do this struggle; therefore, approaching a new concept from different approaches can help reinforce learning and memory (Goel et al., 2010; Jacobson et al., 2016). Their first encounter with new information occurs during the online lecture, the second encounter is in class, which reinforces it several ways. First, by a concept quiz. Second, by instructional activity and, third, by working alone or in groups with problem solving. We believe that this is why many of the struggling students performed better with the multiple approaches/reinforcements found in the flipped format than they would have in just the traditional lecture format. During the flipped section of the course, students were awarded points for participation in quizzes and using iClicker. In the traditional lecture, students could still participate with iClicker to in-class surveys and for some participation points. In lectures that did not utilize iClicker participation points, attendance decreased by 18% as recorded by iClicker attendance as lecture recordings were still made for review.

Is there evidence that flipping the classroom improves student learning and retention? In our survey, this pedagogy was effective for the weaker cohort of students. Others who have performed meta-studies to systematically and quantitatively assess the flipped model argue that student performance improves over the traditional lecture model in several disciplines (Gong et al., 2024; Låg & Sæle, 2019; Strelan et al., 2020; Vitta & Al-Hoorie, 2023). The addition of pre-class, in-class, and post-class material to support a recorded lecture reinforces concepts that are either difficult to understand or that are easily missed in the traditional lecture. Now specifically in the biochemistry subject material, most instructors and researchers have found that there is improved learning, teamwork, and perception of the subject material (Alonso-Chamorro, 2019; Jafarkhani & Jamebozorg, 2020; Schneider et al., 2019; Zaman et al., 2022; Zhang et al., 2020). Not all of these and others report improved final grades for their courses. Anecdotal evidence from this author in discussions with other instructors at national conferences focusing on biochemistry education is that there are many flipped/inverted class failures. Reasons for this can vary from preparation of in-class activity to classroom layout. In supplemental Fig. 2, we provide the in-class material for other instructors to get started. This material is derived from several common textbooks as well as original ideas from the instructors (Fig. S2). Feedback from multiple instructors and students within the discipline suggests that our methodology is more successful than in traditional = based biochemistry classes.

How does technology-based learning (TBL) factor in with the flipped/inverted biochemistry class? We did not incorporate any advanced technology such as virtual reality or animation other than what is provided by iclicker and publisher produced videos. A big advantage to the flipped technique is that there is more time in class to review concepts with any of these technology tools. There are several reports in the literature who all say that the incorporation of TBL has its place and is generally well received. These include animated videos to demonstrate concepts (Wikandari et al., 2021), WeChat platforms (Ji et al., 2023), and experimental simulation platforms such as Icourse and DingTalk (Sun et al., 2023). TBL may be seamlessly incorporated within the classroom activity and teaching to foster greater understanding and reinforcement of difficult concepts.

Some of the weaknesses of this course study are apparent. First, the end-of-course survey was entirely voluntary, in which 54% of the students responded. Students that voluntarily respond tend to be high performing, attention-seeking, or have a strong opinion about the course, whereas those who have a neutral feeling about the course tend not to do any extra work or activity. Yet, our sample size can still give adequate guidance for what did and did not work in this combined flipped/traditional lecture class. Data in the impromptu survey (Fig. 4) aligns well with the end-of-semester survey (Fig. 5 and 6). Second, this study only encompassed one large class in one semester and not several classes over several semesters. Nonetheless, all of our data, both quantitative and anecdotal, can still give guidance on how to improve the course.

## Conclusions

In a content-heavy course such as biochemistry where there are many new concepts and many “pieces” to understand and even memorize, the preferred execution of the course, according to our data, is to flip the class with an online lecture and then activities in class to challenge what the students learned in the online lecture and to reinforce those concepts. Student participation in activities were higher in the flipped course, and by exam 2, the students understood more of the basic concepts compared to the traditional lecture. Going forward, we intend to flip the entire course with a focus on enriched in-class activities to reinforce learning the concepts of basic biochemistry.

## Supplementary Information

Supplementary Material 1

## Data Availability

The datasets used and/or analyzed during the current study are available from the corresponding author on reasonable request.

## Abbreviations

LMS: Learning management system
STEM: Science technology engineering and math
MOOC: Massive open online courses
TBL: Technology-based learning
