# Supplementary material for: Student comprehension of biochemistry in a flipped classroom format
Source: Smart Learn Environ. 2024 Dec 4;11(1):57. doi: 10.1186/s40561-024-00356-z (PMC11698299; doi:10.1186/s40561-024-00356-z)
Supplement: Supplementary file 1 — Supplementary Material 1 [file 40561_2024_356_MOESM1_ESM.pdf]

STUDENT COMPREHENSION OF BIOCHEMISTRY IN A FLIPPED CLASSROOM FORMAT

Edward N. Harris*, Evan A. Schroder, Teryn J. Berks,

University of Nebraska – Lincoln, 1901 Vine St., Lincoln, NE, United States, 68588

Supplemental Fig. 1

Student responses to the question “What advice would you give the instructor to make this course better?”

- Having the flipped classroom setting with class work being done during lecture was helpful.

- In speaking to other students, I've found that most of us find it helpful to learn a small portion of material and then do examples that drive that point how (meaning back and forth between the two multiple times in one class period). The instructor could help their students learn better by providing more example questions and relating them back to specific parts of the material.

- I think the class went pretty well. It definitely has been difficult. I personally enjoyed the flipped format for the class so if the entire semester was like that I would enjoy it!!

- Make the entire class flipped so they we can do the homework in class and ask questions about it.

- I really enjoyed the flipped classroom. It took a lot of time, but it was good to get the general overview of the information and then dive into specifics and practice in class.

- Maybe more of an emphasis on what exactly needs to be known for exams, and making sure to emphasize these points in class, or posting something every lecture that sums up what needs to be known for exams from each lecture.

- I feel like we just dive into the information and are not presented with a broader idea of how things work. I don't think there is enough detail or further explanation on harder concepts, but rather just regurgitating what is on the slides.

- The classwork was extremely helpful as it not only gave us a chance to apply what we're learning to "real" scenarios but it sometimes felt like you were solving them on the spot during class.

- Being able to see how the problems are done really helps when it comes to exams. I felt a lot more confident going into the first and second exam compared to the third exam.

- I surprisingly enjoyed the flipped classroom structure for the first half of the semester. The most beneficial part about it was having the opportunity to work through the classwork assignments with the guidance of Dr. Harris. Seeing problems worked out provided me with the resources to replicate similar problems on the exams. My scores on the first two exams reflected this.

- I preferred the flipped format for days when we covered large amounts of material that didn't involve math or pathways. Having the lecture recordings has been very helpful for me even when class is in person, because I have been re-watching them after class to take more detailed notes. I think that I'd definitely be struggling without online/recorded lectures. I think doing the math portions in person was helpful, since I wouldn't have known how to do some of the earlier classwork if it was just take-home homework unless it was covered in lecture. Sometimes it was still a bit unclear what to do, I remember the acid-base stuff in particular was confusing. On the other hand, having conceptual homework about some of the pathways has been helpful in getting me to think through the process on my own. I think the main difficulty of this class is just the amount of content, and that the exams have been fair based on what was covered in class.

- For the most part I enjoy both the flipped and traditional lectures. At time I feel like certain things should be left off if it's not on the exam but it's great information that does put things in perspective.

- I loved how you had a 50/50 format of flipped and traditional lecturing respectively. The flipped format helped especially with learning the mathematical concepts. For the more context-heavy topics, it made more sense to have a traditional lecture format. My advice would be to do a flipped format for the math-heavy concepts and a traditional format for the concept-heavy topics. The online lectures also were beneficial for me, especially with topics that I found difficult to understand. This allowed me to rewatch the topics that I struggled with and better understand them with more exposure.

- This class was what I expected coming into an upper level biochem course. I generally enjoyed the topics and felt that the exams were manageable. I personally preferred the

flipped classroom method. It was more helpful in understanding the material as we got to watch the lectures beforehand, come in with questions, and be able to do the worksheets as a class. This way prepared me a lot more for what was gonna be on the exams. However, I feel as though the conceptual free response questions on exams were a bit difficult and that they could be covered more on homework and in class. However, everything else was great!

- First of all, I liked the teaching style of the instructor. Personally, it was not an easy course at the beginning but as time passed I was able to catch up because I enjoyed the class. One thing I would advise the instructor is to only use the flipped classroom style because I believe it worked better for most of the students including myself.

- Continue the flipped format.

- Do the lectures on line and homework in class for the entire semester.
- If doing the homework on our own time and lecture in class, maybe make videos about how to do the homework because sometimes it is confusing how the answer was obtained.

- I feel that many times your homework does not represent what we learn in class. Your classwork is more difficult than what we learn from your slides so having you walk through it in the flipped version is more beneficial for me on exams. Because you doing it, teaches me how to do it on exams. Whereas for the exam 3 I had to figure it out myself. I was not a fan of the traditional style.

- I think in class or online lectures could both work, but it seems like the online lectures had a better flow. Maybe this is because it is possible to pause the lecture to digest and take notes. I'm sorry I can't describe why the flow felt better in more detail as I'm not certain as to the exact reason. For the in-class lectures, putting the powerpoints up at least a day in advance would be helpful (especially as the book reading is not required). Going over the math-based material in class has been extremely helpful. Overall, I've been very pleased with the way the class was taught. It has required a lot of study-time outside of class, but that should be expected for a 400 level class.

- I liked the flipped lecture format quite a bit and it helped me learn pretty well. The only thing was that in class quizzes were not given enough time in class and it could be beneficial to be able to use notes or work in a group on those. However, I did like the

flipped format. Having recordings of the lectures in either scenario was super helpful to be able to go back and use as a study tool for exams.

- It may not be possible with the amount of material there is to cover, however I have found that I learn best when I am taking handwritten notes along with the professor during class time. I found it difficult to learn and study using the PowerPoint slides.

- I really enjoyed the class when it was flipped- it was very easy to stop and rewatch sections I didn't understand, and I was better able to take notes which helped me remember it more later on when I was studying for the exams. I would recommend doing the entire class flipped format if possible.

- I unfortunately am taking the course for the second time currently (also enrolled first semester last fall). The flipped lecture made learning the information much more manageable. It did not make the lecture drag on and offered time for you to work through problems for us. Last year, I would show up to exams without knowing how to actually DO any of the questions, despite doing all of the pre-exam prep that you offered. This year during the flipped section I felt like I was actually learning both the content AND the application. Would very much suggest continuing this next year, and I wish there was more room for external supplemental material.

- I find doing the homework on our own time very difficult as we are unsure how to answer some of the questions and have no guidance in doing so. Doing the homework in class is very helpful for learning the material and also understanding how to get the right answer rather than guessing what it is based on the available resources. The math questions specifically there are no examples in the lecture slides which makes it very difficult to complete those without guidance and walking through them in class was very beneficial to understanding material for the exams.

- I feel like the flipped lectures helped me learn more. I liked coming into class having a general idea about the concepts we would be quizzed on. I also really liked the in-class worksheets. The homework has not been really helpful because I don't receive immediate feedback like I did with the in-class worksheets.

- Instructor should encourage students to attend recitals if they have time since there is very limited time during lectures for questions. The in class quizzes should be reviewed since students are on the edge. Technology issues play a role in affecting responses to

the quizzes. Nonetheless, the course has been very transformative and insightful. Current approach has been beneficial to me.

- I think the quizzes could be helpful but more time needs to be given to complete them. They are small points but affect your grade dramatically if you dont get 4's or 5's every time. Also the flipped format had the best results since I could watch the lecture and come prepared to put the concepts I learned to use on the homework with the guidance of the instructor. When doing the homework out of class most it feels alien and asks a lot of deep conceptual questions that are often confusing and hard to see the relevance of concepts we've learned. Better instruction on how to tackle the homework questions could really improve learning in the course and grades on the exams.

- I think doing the split of the half flipped and half traditional class is working well. It is nice to have both of them. The nice part about traditional is that I know what the most important parts of the lecture are to focus on when I'm filling my notes in more and watching the video of class. If lecture videos were not posted with the traditional format, I would do much worse in the class. There is no way for me to take all the notes I need with the amount of information we cover and the speed. Either method, I'm going to be watching the lecture video. With the traditional format I'm exposed the material more because I hear it in class and again when I rewatch the video and again when I am doing the homework. With the flipped format, I'm exposed the material less only the one time when I watch the video and during class when we do the homework together. This seems to be more important with the metabolic pathway parts. I think that the more mathy lectures being flipped is extremely helpful!! I would not have been able to understand the homework as well and I would've gotten stuck, confused, and frustrated more often if we didn't do them together in class.
- Continue with the flipped method

- I did really well during the flipped classroom. I like that I was able to learn the material before class and then attempt homework problems in class. If I had any issues with the homework or understanding it, the time in class could be used to resolve these issues.

Supplemental Figure 2

Lesson #1 pH, pKa, and water

Identify the conjugate acids and bases of the following pairs

$^{+}H_3NCH_2COOH/^{+}H_3NCH_2COO^{-}$

$^{+}H_3NCH_2COO^{-}/H_2NCH_2COO^{-}$

The equilibrium constant K_{eq} for the reaction A ←→ B is $2.6x10^4$ at 25°C. What is ΔG° for this reaction at equilibrium?

ΔG°= -RTlnKeq

= -(8.3x10x10^{-3} kJ/mol K)(298 K)ln2.6x10^4

= -25.1 kJ/mol

What is the equilibrium constant if the ΔG° is nearly zero at -0.1 kJ/mol?

-0.1 kJ/mol = -(8.3x10x10^{-3} kJ/mol K)(298 K)

-0.1 kJ/mol/-(8.3x10x10^{-3} kJ/mol K)(298 K) = 0.0404 = lnKeq which is $e^{0.0404}$ = 1.04

Is this reaction favorable/spontaneous?

Yes for the first and barely yes for the second

What is the ratio of the conjugate base to acid in a weak acid with pKa of 4.2 at a pH of 6.6?

pH = pKa + log(A/HA) 6.6 = 4.2 + log A/HA

2.4 = Log A/HA

$10^{2.4}$= A/HA

251 = A/HA

What is the pH of a solution that has a H^+ concentration of: pH = -log[H+]

a. 3.1x10^{-6} M 5.5

b. 9.1x10^{-5} M 4.0

c. 4.4x10^{-2} mM 4.4

d. 1.7x10^{-4} uM 9.8

What are the concentrations of H+ and OH- in a solution of 120 mM NaOH?

$Kw = [H+][OH-] = 1.0x10^{-14}\ M^2$, $[OH-] = 0.12M$

$[H+]=1.0x10^{-14}\ M^2/0.12M = 8.3x10^{-14}\ M$

Calculate the pH of a dilute solution that contains a molar ratio of potassium acetate to acetic acid (pKa = 8.2) of use pH = pKa + log[A-]/[HA]

a) 1:5 7.5

b) 1:30 6.7

c) 22:3 9.1

Calculate the pH of a buffer solution that contains 0.1 M acetic acid and 0.25 M sodium acetate (pKa=7.6).

pH= 7.6 + log (0.25/0.1)

8.0

A lab recipe gives direction on making 1 liter of Tris Buffer at 50 mM, pH 8.0. using 2.02 g TRIS free base (121.1 g/mol) and 5.25 g of TRIS HCl (157.6 g/mol). Verify if this is correct.

2.02 g/121.1 g/mol = 0.0167 mol

5.25g/157.6 g/mol = 0.033 mol: sum of these 2 is 0.05 mol but check with Henderson-haseelback

pH = 8.3 + log(0.0167/0.033) = 8.0

If you mix equal volumes of 0.1 M HCl and 0.2 M TRIS (pKa 8.3), is this solution a buffer?

Due to equal volumes the molarities are cut in half, 0.05 M HCl and 0.1 M TRIS. In the reaction, you end up having 0.05 M protonated TRIS and 0.05 M TRIS free amine. Yes this is a buffer.

What is the pH of this solution? pH = 8.3 + log (0.05/0.05) = 8.3

If you have 100 mL of 0.1 M TRIS buffer at pH 8.3 and you add 3.0 mL more of 1 M HCl, what is the new pH? Both the acid and conjugate base are each at 0.05 M, account for volume and that is 0.005 M pH = 8.3 + log (0.002/0.008) skew each side by 0.003 = 7.7

What is pH if you add another 3 mL? pH = 8.3 + log(0.002-0.003/0.008+0.003) We lose the buffering capacity of the conjugate base so now we just have pH = -log (0.01)= 2 (0.001M in 0.1 L of solution)

Lesson #2 protein chemistry

1. Calculate the charge of the following amino acids at the given pH values.

	pKa	pH 2	pH 4.5	pH 6.8	pH 9	pH 11

Alanine	--	+1	0	0	0	-1
Aspartate	4	+1	-1	-1	-1	-2
Arginine	12.5	+2	+1	+1	+1	0
Lysine	10.5	+2	+1	+1	+1	-1
Cysteine	8.3	+1	0	0	-1	-1
Tyrosine	10.1	+1	0	0	0	-2
Histadine	6.1	+2	+1	0	0	-1

2. (3 pts) Calculate the charge of the following peptide: A-R-E-L-S-P-D-Q-H-V. Use information in the textbook or lecture slides for amino acid pKa values.

At pH 3 NH3+ = +1, R = +1, E = 0, D = 0, H = +1, COO- = -1 → 2

At pH 7 NH3+ = +1, R = +1, E = -1, D = -1, H = 0, COO- = -1 → -1

At pH 11 NH3+ = 0, R = +1, E = -1, D = -1, H = 0, COO- = -1→ -2

Lesson #3 Protein Folding

Hair grows at a rate of 15 to 20 cm/yr. All this growth is concentrated at the base of the hair fiber, where a-keratin filaments are synthesized inside living epidermal cells and assembled into ropelike structures. The fundamental structural element of a-keratin is the a helix, which has 3.6 amino acid residues per turn and a rise of 5.4 Å per turn. Assuming that the biosynthesis of a-helical keratin chains is the rate-limiting factor in the growth of hair, calculate the rate at which peptide bonds of a-keratin chains must be synthesized (peptide bonds per second) to account for the observed yearly growth of hair.

Answer Because there are 3.6 amino acids (AAs) per turn and the rise is 5.4 Å/turn, the length per AA of the a helix is 5.4 A/turn divided by 3.6 aa/turn

=1.5 Å/AA = 1.5 x 10^{-10} m/AA

A growth rate of 20 cm/yr is equivalent to 20 cm/yr divided by (365 days/yr)(24 hr/day)(60 min/hr)(60s/min)
= 6.3 x 10-7 cm/s =6.3 x 10-9 m/s

Thus, the rate at which amino acids are added is 6.3x10-9 m/s divided by 1.5x10-10 m/aa
= 42 AA/s = 42 peptide bonds per second

Which of the following peptides is more likely to take up an helical structure, and why?
(a) LKAENDEAARAMSEA
(b) CRAGGFPWDQPGTSN

Answer By cursory inspection, peptide **(a)** has five Ala residues (most likely to take up an
_-helical conformation), and peptide **(b)** has five Pro and Gly residues (least often found in an
_ helix). This suggests that **(a)** is more likely than **(b)** to form an _ helix.

Glycine is a highly conserved amino acid in proteins. Why????

Answer: Because it is involved with b-turns and folding and conserved proteins have the same shape.

A mutation that changes an alanine residue in a protein to an isoleucine leads to a loss of function. Activity is regained when a further mutation at the same site changes the isoleucine to a glycine. Why?

Iso is bulky and interferes with function whereas glycine is not.

Replacement of W causes the greatest effect on protein structure and function.

Big and hydrophobic so it will disrupt the 3D structure

Replacements such as K→R and L→I usually have very little effect on protein structure and function.

They are similar amino acids.

Why does wool tend to shrink when washed and silk does not???

Wool is keratin that is rich in a-helicies so there is shrink and stretching occurring with the helix. Silk is mostly b-sheets which do not stretch the same.

Name 4 types of noncovalent interactions that stabilize tertiary and quaternary structures.

Ionic, hydrogen, van der waals, hydrophobic

The amino acid residues in protein molecules are exclusively L stereoisomers." It is not clear whether this selectivity is necessary for proper protein function or is an accident of evolution. To explore this question, Milton and colleagues (1992) published a study of an enzyme made entirely of D stereoisomers. The enzyme they chose was HIV

protease, a proteolytic enzyme made by HIV that converts inactive viral preproteins to their active forms.

Previously, Wlodawer and coworkers (1989) had reported the complete chemical synthesis of HIVprotease from L-amino acids (the L-enzyme). Normal HIV protease contains two Cys residues at positions 67 and 95. Because chemical synthesis of proteins containing Cys is technically difficult, Wlodawer and colleagues substituted the synthetic amino acid L-a-amino-*n*-butyric acid (Aba) for the two Cys residues in the protein. In the authors' words, this was done to "reduce synthetic difficulties associated with Cys deprotection and ease product handling."

The structure of Aba is shown below. Why was this a suitable substitution for a Cys residue? Size and charge issues

Under what circumstances would it not be suitable? Cannot do a disulfide bond.

Wlodawer and coworkers denatured the newly synthesized protein by dissolving it in 6 M guanidineHCl, and then allowed it to fold slowly by dialyzing away the guanidine against a neutral buffer(10% glycerol, 25 mM NaPO4, pH 7).

(b) There are many reasons to predict that a protein synthesized, denatured, and folded in this manner would not be active. Give three such reasons.

There are many important differences between the synthesized protein and HIV protease
produced by a human cell, any of which could result in an inactive synthetic enzyme: (1) Although Aba and Cys have similar size and hydrophobicity, Aba may not be similar enough for the protein to fold properly. (2) HIV protease may require disulfide bonds for proper functioning. (3) Many proteins synthesized by ribosomes fold as they are produced; the protein in this study folded only after the chain was complete. (4) Proteins synthesized by ribosomes may interact with the ribosomes as they fold; this is not possible for the protein in the study. (5) Cytosol is a more complex solution than the buffer used in the study; some proteins may require specific, unknown proteins for proper folding. (6) Proteins synthesized in cells often require chaperones for proper folding; these are not present in the study buffer. (7) In cells, HIV protease is synthesized
as part of a larger chain that is then proteolytically processed; the protein in the study was synthesized as a single molecule.

(c) Interestingly, the resulting L-protease was active. What does this finding tell you about the role of disulfide bonds in the native HIV protease molecule? The disulfide bond is not really needed.

Lesson #4 Protein Function

1. (3 pts) A human buccal cell contains 1800 aquaporin structures in the plasma membrane. If water flows through these channels at 1.75×10^6 molecules per second per aquaporin, and the volume of the buccal cell is 6×10^{-10} mL, how rapidly

would the cell lose 1/4 of its volume in the presence of high salt (McDonald's fries)? Water density is 55.5 mol/L., Avagadro's number is 6.02 x 10^{23} molecules/mol. Hint: figure out number of water molecules occupying 1/4 of the cell volume and the rate in which the water molecules leave the cell.

To get number of water molecules going out of cell:

1800 aquaporins x $1.75 \times 10^6 = 3.15 \times 10^9$ water molecules/sec

To get 1/4 volume of cell: $6 \times 10^{-10} \times 0.25 = 1.5 \times 10^{-10}$ mL or 1.5×10^{-13} L

To get number of molecules occupying 1/4 cell volume

55.5 mol/L x 1.5×10^{-13}L x 6.02×10^{23} molecules/mol = 5×10^{12} molecules

To get the rate in seconds: 5×10^{12} molecules/3.15×10^9 molecules/sec = 1590 seconds

The concentration of fructose in the blood is about 0.1 mM. What is the free energy required to transport it across a membrane of 0.06 V into a cell with an internal concentration of 0.035 mM fructose.

G=RTlnC2/C1+ZFV Since fructose is neutral, Z=0

G=(0.0083 kJ/mol K)(310 K)ln(0.035/0.1) = -2.7 kJ/mol, yes it is favorable

Bioenergetics: The sodium/potassium pump in a nerve cell (ATPase) pumps out 3 sodium ions and pumps in 2 potassium ions. The membrane potential is -70 mV and the inside of the cell is negative relative to the outside of the cell. The ion concentration is as follows

Potassium: 5 mM outside, 140 mM inside

Sodium: 10 mM inside, 150 mM outside

What is the energy requirement to transport 2 moles of potassium across the membrane?

G=(0.0083 kJ/mol K)(310 K)ln(140 mM/5 mM)+(1)(96.5 kJ/mol V)(-.07 V) = 1.82 kJ mol then multiply by 2 for 2 moles. (electrically favorable, not energetically favorable) 8.57+(-6.76)=1.8 kJ/mol then multiply by 2 = 3.6 kJ

What is the energy requirement to transport 3 mols of sodium across the membrane?

G=(0.0083 kJ/mol K)(310 K)ln(15×10^{-2} M/1×10^{-2} M)+(1)(96.5 kJ/mol V)(0.07 V) = 13.7 kJ/mol then multiply by 3 for 3 moles (both not electrically or energetically favorable). [charge is reversed when pumping outside the cell] 41.1 kJ

Magnesium is transported across the membrane by MAGT1 transporter. The [magnesium] is 5 uM outside the cell and 1 uM inside the cell in humans. What is the free energy change to get magnesium inside the cell if the membrane potential is 0.05 V?

G=8.3x10-3KJ/mol K * 310K ln(1/5) + 2(96.5 kJ/mol V)(-0.05 V) = 9.0 kJ/mol

G = -4.14 kJ/mol - 9.65 kJ/mol = -13.8 kJ/mol It is negative because we are going into a negative environment

Calculate the energy required to pump glucose out of the cell using a glucose-sodium symporter of an epithelial cell when Na-in is 12 mM, Na-out is 145 mM and the membrane potential is -50 mV, temp is 37 C. 2 moles of Na is required to move 1 mole of Glc.

G=8.3x10-3 kJ/mol K (310K) ln (0.145/0.012) + 1(96.5 kJ/mol V)(0.05 V) charge is positive because outside of cell is positive. 11.2 kJ/mol for 1 mol of Na so 11.2 kJ/ml x 2 = 22.4 kJ

What is the amount of energy required to pump 1 mol glucose given that 2 mols of sodium are required to pump 1 mol glucose?
11.2 kJ/mol x 2 = 22.4 kJ/mol

What is the maximum concentration of glucose that can be achieved by this pump?
Use G=RTlnK

22.4 kJ/mol = 8.3x10-3kJ/mol K(310K)ln (glucose out/glucose in)

Solve for the ratio which is 8.7 and do inverse ln e^8.7 is 6037 or about 6000 times more glucose out the cell than in the cell.

A human blood cell has about $2x10^3$ aquaporin A monomers. If water molecules flow through the plasma membrane at a rate of $5x10^8$ per aquaporin tetramer per second, and the vol of the blood cell is $5x10^{-11}$ mL, how rapidly could a blood cell halve its volume as it encountered the high osmolarity in the interstitial fluid of the renal medulla? Assume the blood cell is all water.
First calculate the number of water molecules that leave the blood cell to halve the volume.
5x10-14 L/cell (6.02x10^23 molecules/mol)(55.5 mol H2O/L = 1.7 x 10^12 molecules and half is 8.5x10^11
Second, how fast can the cell lose water
2x10^3 x 0.25 = 500 tetramers that allow 5x10^8 molecules per second
5x10^8 H2O/sec/tetramer (500 tetramers/cell = 2.5 x 10^11 H2O/sec
8.5x10^11 H2O/2.5x10^11 H2O/sec = 3.4 sec

Lesson #5 Methods I

Table: Isoelectric points of several common proteins

Protein	Isoelectric pH
Pepsin	0.7
Ovalbumin	4.6

Serum albumin	4.9
tropomyosin	5.1
Insulin	5.4
Fibrinogen	5.8
Gamma-globulin	6.6
Collagen	6.6
Myglobin	7.0
Hemoglobin	7.1
Ribonuclease A	7.8
Cytochrome C	10.6
Histone	10.8
Lysozyme	11.0
Salmine	12.1

Table 1: In what order will the following proteins be eluted from CM-cellulose ion exchange column by an increasing salt gradient at pH 7: fibrinogen, hemoglobin, lysosome, pepsin and ribonuclease A?

Answer: Pepsin, fibrinogen, hemoglobin ribonuclease A, lysosome

Table 1 What would be the arrangement of the following proteins after isoelectric focusing: insulin, cytochrome C, histone, myglobin, and ribonuclease A?

Answer: Insulin, myglobin, ribonuclease A, cytochorome C, histone.

Table 2

Protein	Molecular Mass (kD)	Partial Specific Volume, $\bar{V}_{20,w}$ ($cm^3 \cdot g^{-1}$)	Sedimentation Coefficient, $s_{20,w}$ (S)	Frictional Ratio f/f_0
Lipase (milk)	6.7	0.714	1.14	1.190
Ribonuclease A (bovine pancreas)	12.6	0.707	2.00	1.066
Cytochrome *c* (bovine heart)	13.4	0.728	1.71	1.190
Myoglobin (horse heart)	16.9	0.741	2.04	1.105
α-Chymotrypsin (bovine pancreas)	21.6	0.736	2.40	1.130
Crotoxin (rattlesnake)	29.9	0.704	3.14	1.221
Concanavalin B (jack bean)	42.5	0.730	3.50	1.247
Diphtheria toxin	70.4	0.736	4.60	1.296
Cytochrome oxidase (*P. aeruginosa*)	89.8	0.730	5.80	1.240
Lactate dehydrogenase H (chicken)	150	0.740	7.31	1.330
Catalase (horse liver)	222	0.715	11.20	1.246
Fibrinogen (human)	340	0.725	7.63	2.336
Hemocyanin (squid)	612	0.724	19.50	1.358
Glutamate dehydrogenase (bovine liver)	1015	0.750	26.60	1.250
Turnip yellow mosaic virus protein	3013	0.740	48.80	1.470

Table 2 Estimate the molecular mass of an unknown protein that elutes from a Sephadex G-50 column between cytochrome C and ribonuclease A.

Answer: 13 kDa

Table 2 What is the order of elution of the following proteins froma Sephadex G-50 column: catalase, chyrotrypsin, concanavalin B, lipase, and myoglobin?

Answer: Catalase>concavalin B>chymotrypsin>myoglobin>lipase

In what order will the following amino acids be eluted from a column of P-cellulose (P=phosphate groups, Negative charge) ion exchange resin by a buffer at pH 6: arginine, aspartic acid, histidine, and leucine

Answer: aspartate, leucine, histidine arginine. At pH 6, arginine has a charge of +1, aspartate -1, histidine has a charge of +1/2, leucine is 0. The resin will bind cations (positive charge things).

A mixture of amino acids consisting of R, C, E, H, L, and S is applied to a strip of paper and subjected to electrophoresis using a buffer at pH 7.5. What are the directions of migration of these amino acids and what are their relative mobilities?

Answer: R will move rapidly to the cathode (-). E will migrate toward the anode (+) with E moving faster than C. H, L, C and S will not move.

What is the molecular mass of a protein that has a relative electrophoretic mobility of 0.5 in an SDS-PAGE in the following regression line?

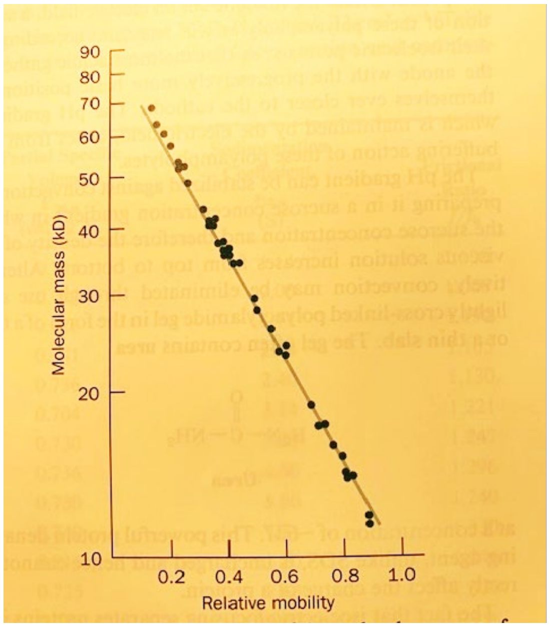

Answer 28kDa

At a pH equal to the isolectric point of alanine (6.01), the net charge on alanine is zero. What are the 2 forms of alanine at a charge of zero?

Answer: zwitterion and completely uncharged.

Why is alanine predominantly switterionic rather than completely unchaged at its pI?

Answer: pKa of the amino group and carboxy group cancel each other out.

What fraction of alanine is in the completely uncharged form at its pI?

Answer: The pI is the midpoint between 2.3 and 9.7 which are pKa of the amino and carboxy groups Use herderson-hasselbalch equation in which pH-pKa=Log(A-)/(HA)

Log A/HA=6.01-2.3 = 3.71

A/HA=10^3.71 = 5129/1 or 1 molecule of protonated alanine is equal to 5129 molecules of deprotonated alanine. For both groups (NH2 and COOH in uncharged forms at pH 6.01 (at pI) then it is 5129^2 = 2.6x10^7 or 1 out of 26 million molecules.

Lesson 6 Methods II

You would like to purify mitochondria from CHO cells in culture. What methods would you use in the following order?

1. Dounce homogenizer, preparative centrifugation
2. Sonication, preparative centrifugation, dialysis
3. French press, preparative centrifugation
4. Dounce homogenizer, size exclusion chromatography?

You are purifying a protein that is purported to be 8 kDa in mass from Fruit fly eyes. After homogenizing the eyes, you centrifuge out the cell debris and end up with a protein solution. Next, you separate the proteins by size exclusion chromatography using Sephadex G25 (anything larger than 10 kDa is in the void volume) and get out the small stuff. You perform dialysis with tubing of 3 kDa pore size. What happened to your protein?

You injected heparin polymers with a tritium tag in a rat and you would like to assess how much ends up in urine over a 12 hour time period. After place the rat in a metabolic cage, you are able to collect urine every hour with different volumes. How would you quantify the heparin in that urine?

Methods II

Step	Procedure	Total protein (mg)	Activity (units)	Specific Activity
1	Crude extract	10,000	20,000,000	2000
2	Precipitation (salt)	9,000	12,000,000	1333.3
3	Precipitation (pH)	7,500	11,000,000	1466.6
4	Ion-exchange chromatography	2,500	5,000,000	2000
5	Affinity Chromatography	2,000	4,000,000	2000
6	Size-exclusion chromatography	250	2,000,000	8000

From the information in the table, calculate the specific activity of the enzyme after each purification procedure and complete the right column of the table. Use this table to answer questions 2-4.

2. (1 pt) Which of the purification procedures used for this enzyme is the most effective and why? Size exclusion Chromatography because there is a 4-fold increase in specific activity.

3. (1 pt) Which of the purification procedures is the least effective and why? salt precipitation because there was a loss in specific activity

4. (1 pt) After step 6, how would you assess if you have pure protein? Take a portion of the protein sample and run it out by SDS-PAGE and stain the gel with Coommassie or silver staining.

6. (2 pt) You discover a new protein and you would like to know if it is a promotor or repressor (binds DNA) based on its protein sequence. What method would you use and why? Use the Luciferase assay. The DNA sequence for the protein can be used to drive Luciferease transcription/translation and give a visual readout.

- A mixture of blue dextran, cytochrome *c*, cobalamin, and DNP-glycine is placed in a Sephadex G25 column (SEC limited to 10kDa). Using the table below, what is the elution order of the colors eluted from the column?

  A. blue, brown, orange

  B. blue, brown, red, yellow

  C. yellow, red, brown, blue

  D. none of the above

Component	MW (kDa)	Color
Blue dextran	50,000	blue
Cytochrome C	12,400	brown
Cobalamin	1,355	red
DNP-glycine	241	yellow

- Dialysis is frequently used in persons with kidney dysfunction to remove waste products from the body. Based on size, which products do you think will not be filtered out through dialysis?

    A. Glucose

    B. Protein

    C. Ammonia

    D. Oxygen

Protein	Subunit mass (Da)	Native mass (Da)	pI
A	10,000	40,000	8.1
B	15,000	120,000	5.3
C	20,000	20,000	7.2
D	25,000	75,000	4.5
E	30,000	60,000	6.2

1. Which protein would elute last from a gel filtration chromatography column under non-denaturing conditions?
2. Which protein would migrate the slowest in an SDS-PAGE gel?
3. Which protein would elute last from an anion-exchange column (DEAE) using a buffer at pH 6.5?
4. What is the likely quaternary structure for the protein D on the basis of the data in the table?

C

E

D

Trimer

What is the best method for the Covid-19 antigen test? Briefly describe how this method works. ELISA assay. It is an antibody-based test in which the antigen bait (viral particles) are used to determine if the patient (you) have antibodies present in your blood. The antigen is part of the assay and antibodies from your blood are stuck to these antigens and then secondary antibodies detect your antibodies and give the signal via a readout enzyme.

Consider the following peptide: H3N-L-S-P-R-W-T-A-A-N-K-I-G-G-F-S-A-R-I-Y-C-G-K-M-V-K-E-C-V-A-T-COOH

Write the amino acids/peptides that are present after cleavage with Trypsin

L-S-P-R, W-T-A-A-N-K, I-G-G-F-S-A-R, I-Y-C-G-K, M-V-K, E-C-V-A-T

Write the amino acids/peptides that are present after cleavage with chymotrypsin

L-S-P-R-W, T-A-A-N-K-I-G-G-F, S-A-R-I-Y, C-G-K-M-V-K-E-C-V-A-T

You compare the proteins from blood samples from 2 siblings; one with blond hair and the other with black hair, by 2D gel electrophoresis. What method would you use to identify unique proteins on the gel. Why? Mass spectrometry as it gives identification of small samples sizes.

What method would you use to visualize the GroEL/GroES complex? Why? Cryo-EM. It is the only method to capture images of large protein complexes that are otherwise to complex or big to determine by other methods such as NMR or Co-IP.

Lesson #7 Enzyme Mechanisms

1. Which of the following effects would be brought about by any enzyme catalyzing the simple reaction?

S→P (k1) S←P (k2) where Keq = [P]/[S]

a. Decreased Keq
b. Increased k1
c. Increased Keq
d. Increased ΔG of entire reaction
e. Decreased ΔG of uncatalyzed reaction
f. More negative ΔG'°
g. Increased k2

b, e, g. Enzymes do not change a reactions equilibrium constant and catalyze the reaction in both directions making **b** and **g** correct. Enzymes increase the rate of a reaction by lowering the activation energy so **e** is correct.

2. Which graph shows a ΔG value that is positive? B

Which graph shows an exogonic reaction? A

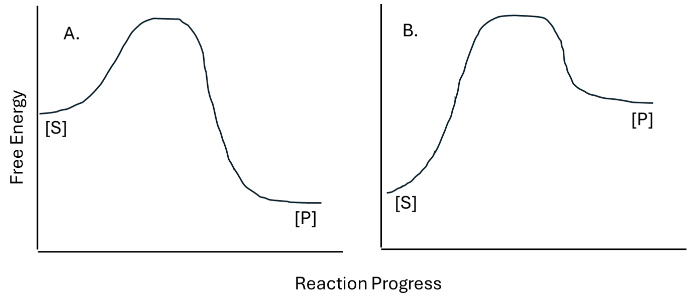

3. For the reaction, X ←→ Z, the forward rate constants are 4.7 x 10^5/s with enzyme and 1.9 x 10^2/s without enzyme. The reverse rate constant is 1.3 x 10^2/s with enzyme. What is the equilibrium constant without enzyme?

Keq = Kf/Kr

4.7 x 10^5/s /1.3 x 10^2/s = Keq = 3.6x10^3

What is the reverse rate constant for the uncatalyzed reaction?

Kr = 1.9x10^2/s/3.6x10^3 = 5.3x10^{-2}/s

What is enhancement rate with enzyme?

Enhancement rate = Kf (cat)/Kf (uncat) = 4.7 x 10^5/s /1.9 x 10^2/s = 2474 fold

4. The equilibrium constant K_{eq} for the reaction A ←→ B is 1.2x10^3 at 25°C. What is ΔG°' for this reaction at equilibrium?

ΔG°' = -RTlnKeq

-(8.3x10^3 kJ/mol K)(298 K)ln1.2x10^3

= -17.6 kJ/mol

4b. Applying the above reaction to the cell (37°C), the actual concentration of A is 0.04 M and the concentration of B is 0.008 M. What is ΔG for the reaction?

ΔG= ΔG°' +RTlnQ

= -17.6 kJ/mol + (8.3x10^3 kJ/mol K)(310 K)ln (0.008/0.04)

= -21.7 kJ/mol

5. The enzyme, urease, enhances the rate of urea hydrolysis at pH 8.0 and 20 C by a factor of 10^9. If a given quantity of urease can completely hydrolyze a given quantity of area in 5 min. at 20 C and pH 8.0, how long would it take for this amount of urea to be

hydrolyzed under the same conditions in the absence of urease? Assume sterile conditions.

Time to hydrolyze urea: 5 min x 10^9/[60 min/hr x 24 hr/day x 365 day x year] = 9513 years

6. An enzyme in bacteria living in a hot springs enhances product formation at 73° C when compared to the same conditions at 25° C. The enhancement rate is by a factor of 7.1×10^5. If this enzyme can synthesize one mole of product in 13.4 hrs at 73° C, how long will it take the enzyme to produce the same amount of product at 25° C?

13.4 hrs x 7.1×10^5 = 9.5×10^6 hrs or 3.96×10^5 days or 1086 years

The addition of magnesium speeds up the reaction at 2.8x the rate at 73° C. How much product can be made in 4 hours? 1 mole made in 4.8 hrs (13.4 hrs/2.8) so 1 mol/4.8 hrs = xmol/4 hrs, x = 0.83 mol

7. In the following reaction, Kf (uncat) is 10^-5/s and Kr (uncat) is 10^-2 and Kf(cat) is 10^7/s. What is Keq, Kr(cat) and rate enhancement?

Keq= Kf/Kr 10^-5/10^-2 = 10^-3 which favors reactants or substrate
Kr = Kf/Keq so 10^7/10^-3 = 10^10/s
Rate enhancement = Kf cat/Kf uncat = 10^7/10^-5 = 10^12 fold

8. Suppose that in the absence of enzyme, the forward rate constant (Kf) for the conversion of S into P is 10^{-4}/sec. and the reverse rate constant (Kr) is 10^{-6}/sec.

A. What is the equilibrium for the reaction?

Keq = $10^{-4}/10^{-6}$ = 100, ΔG°'= -RTlnKeq, = -(8.3x10-3 kJ/mol)(298 K)ln100 = -11.7 kJ/mol

8B. Suppose an enzyme enhances the rate of the reaction 100-fold. What are the rate constants for the enzyme-catalyzed reaction? What is the equilibrium constant? What is the ΔG°'?

The new Kf and Kr are 10^{-2}, and 10^{-4}, respectively. ΔG°' does not change. Nor does the Keq.

9. What would be the result of an enzyme having a greater binding energy for the substrate than for the transition state?

No catalysis since the reaction will not proceed forward.

10. Consider the following reaction: G-6-P ←→ G-1-P. After reactant and product were mixed and allowed to reach equilibrium at 25 C, the concentration of each compound was measured.

G-1-P=0.01 M

G-6-P=0.19 M

Calculate Keq and ΔG°'

Keq = 0.19/.01 = 19

ΔG°' = -8.3x10-3 (298) ln 19 = -7.3 kJ/mol

Lesson #8 Hemoglobin

Why do we have red blood cells and not just free Hb in our plasma?

A. red blood cells prevent Hb from soaking into the tissues

B. Hb works better in high concentrations within cells

C. High protein concentrations would destroy the vascular system

D. Everyone would have a reddish tint.

1. (2 pts) In the following graph the solid line represents hemoglobin that is in blood that has recently left the heart and is on its way to the capillary beds of the tissue. Label the lines for the following: A) Hemoglobin in very low pH (6.9), B) Hemoglobin in venous blood, C) Hemoglobin in the fetus. What is the mm Hg at θ_{50} value for fetal hemoglobin if the material hemoglobin is at 60 mm Hg for θ_{50}?

A = dash/dotted line
B = dashed line
C = dotted line
fHb is ~40 mm Hg

(½ point for each)

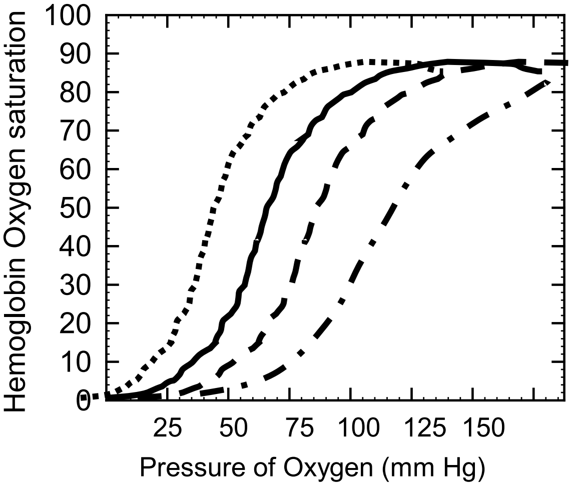

2. (2 pts) A team of UNL SBS professo[illegible] squirrel that produces hemoglobin as a dimer instead of the normal tetramer. Is this new hemoglobin going to bind oxygen with greater or lesser affinity compared to hemoglobin in natural squirrels? If both the genetically engineered and normal squirrels are running on a treadmill at an elevation of 4000 meters, which one will be exhausted first and why?
Dimeric hemoglobin will bind oxygen with greater affinity, akin to myoglobin. The genetically altered squirrel will be exhausted first because its hemoglobin cannot off-load oxygen as well as the natural squirrel and become hypoxic.

Review high elevation, bohr effect, gamma globin

3. What is the effect of the following changes on oxygen affinity of Hb?

A. A drop in the pH of blood plasma from 7.4 to 7.2 decrease

B. A decrease in the partial pressure of CO_2 in the lungs from 6 kPa to 2 kPa? increase

C. An increase in the BPG level from 5 mM to 8 mM? decrease

D. An increase in CO from 1 ppm to 30 ppm? None to increase

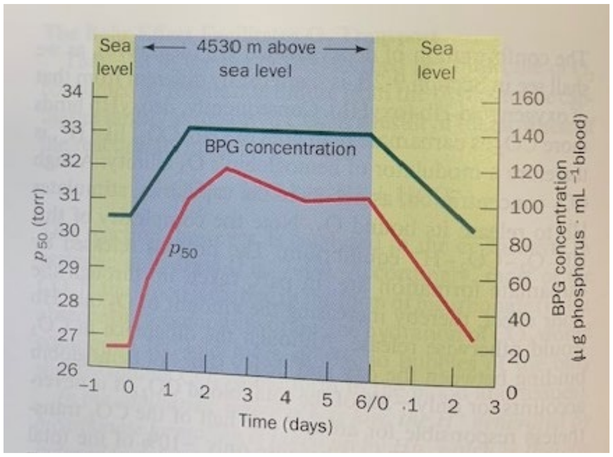

As one of the favorites to win the La Paz, Bolivia marathon, you have trained there for the several weeks it requires to become acclimated to the 3700 m altitude. A manufacturer of running equipment who sponsors an opponent has invited you for the weekend to a pre-race party at a beach house near Lima, Peru with the assurance that you will be flown back to La Paz at least a day before the race. Is this a token of his respect for you or an underhanded attempt to handicap you in the race?

The 48 hr stay at sea level after adaptions lower the p50 and BPG beyond the ability of a 24 hr recovery.

The urge to breathe results from high blood CO_2 content; there are no direct physiological sensors of blood pO_2. Skindivers often hyperventilate just before making a protracted dive in the belief that they will thereby increase the oxygen content of their blood. This belief results from the fact that hyperventilating represses the breathing urge by expelling significant quantities of CO_2 from the blood. In light of what you know about the properties of hemoglobin, is hyperventilation a useful procedure?

Since arterial blood in a normal individual at sea level is nearly fully saturated with oxygen, hyperventilation can have little direct effect on the oxygen content of blood. However, excessive removal of CO_2 would consume H^+ in the reaction

$H^+ + HCO_3^- \leftrightarrow H_2O + CO_2$

The resultant higher blood pH would increase the O_2 affinity of Hb through the Bohr effect so that less than normal amount of oxygen would be delivered to the tissues until the CO_2 balance was restored. Thus, hyperventilation might have an opposite effect to what was intended. Furthermore it is a dangerous procedure because repressing the breathing urge by lower the CO content of the blood may cause the diver to loose consciousness due to the lack of oxygen and may drown.

Erythrocytes that have been stored for over a week in standard acid-citrate-dextrose medium become depleted in BPG. What are the merits of using fresh versus week-old blood in blood transfusions?

Depletion of BPG shifts Hb's fraction saturation curve to the left so that the amount of oxygen that Hb can deliver to the tissues is reduced.

An anemic individual, whose blood has only half the normal Hb content, may appear to be in good health. Yet, a normal individual is incapacitated by exposure to sufficient carbon-monoxide to occupy half his heme sites (CO binds to heme at 200x than oxygen). Why is the anemic person still alive?

In anemia, the Hb that is present functions normally and is present in sufficient quantity to carry the required amount of oxygen. In the CO poisoning case, half the oxygen binding sites of Hb bind CO essentially irreversibly. This converts most of the Hb to the R state which greatly increases its oxygen affinity over that of normal Hb. Little of the oxygen carried by this Hb will be discharged resulting in asphyxiation.

The crocodile, which can remain under water without breathing for up to 1 hr, drowns is pretty and eats it. An adaption that aids the crocodile in doing so is that it can utilize virtually 100% of the oxygen in its blood, whereas humans can extract only ~65% of the oxygen in their blood. Crocodile Hb does not bind BPG. However, crocodile deoxyHb preferentially binds HCO_3^-. How does this help the crocodile obtain dinner?

As the croc remains underwater without breathing, it generates HCO_3^- in the blood. HCO_3^- preferentially binds to the deoxyHb, which allosterically, prompts the Hb to assume the deoxy conformation and release its oxygen.

The severely anemic condition of homozygotes for HbS (sickle cell disease) results in an elevated BPG content in their erythrocytes. Is this beneficial?

Delivery of oxygen is increased, however, loading of oxygen is decreased and the sickle shape of the RBC aggravates the disease. It's a mixed blessing.

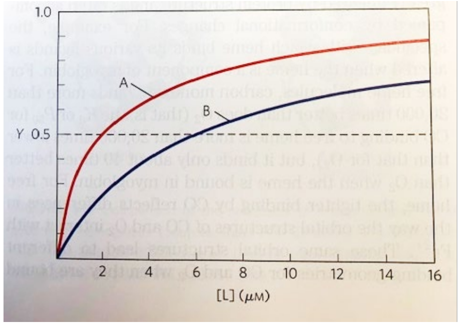

Which of these proteins has the highest affinity for ligand (L)?

A. A red

B. B blue

What is the dissociation constant for protein A?

A. 0.5 uM
B. 2 uM
C. 6 uM
D. Cannot be determined

Lesson #9 Enzyme kinetics I

1. An enzyme catalyzed reaction has a Km of 1 mM and a Vmax of 5 nM/s. What is the reaction velocity when the substrate concentration is

a) 0.25 mM, b) 1.5 mM, and c) 7.0 mM?

Vo= (5x10^-6 mM/s)(0.25 mM)/ 1 mM + 0.25 mM

Vo = 1 nM/s, 3 nM/s, 4.8 nM/s

2. An enzyme is found that catalyzes the reaction A ←→ B. Researchers find that the Km for the substrate [S] is 4 uM and the kcat is 20/min

In an experiment, [S] = 6 mM and Vo = 480 nM/min. What is the [Et] used in the experiment?

Find Vmax first 480 nM/min = Vmax(6 uM)/(4 uM+6 uM)

480nM/min/Vmax = 6 uM/10 uM

Vmax = 0.8 uM/min or 800 nM/min

Kcat=Vmax/Et so 20/min = 800 nM/min/Et solving for Et is 40 nM

3. An enzyme catalyzes the reaction C ---> D. The enzyme is present at a concentration of 1.3 nM, and the Vmax is 7.1 mm/s. The K_m for substrate A is 11 mM. Calculate the initial velocity of the reaction, V_o, when the substrate concentration is 3 uM.

Vo=Vmax[S]/Km+[S]

Vo = 7.1 uM/s[3 um]/11 uM + 3 uM = 1.5 uM/s

4. A variant of the enzyme catalyzes the reaction C ---> D. This enzyme is present at a concentration of 5.3 nM, and the Vmax is 18.2 uM/s. Calculate the Km for the reaction when the substrate concentration is 2.5 uM. Assume Vo is 1/2 of Vmax.

9.1 uM/s = 18.2 uM/s(2.5 uM)/(Km + 2.5 uM

9.1 uM/s(Km) + 18.2 uM2/s = 45.5 uM2/s

9.1 uM/sKm = 27.3 uM2/s

Km = 3 uM

5. An enzyme, Murinase, converts X ↔ Y with a Kcat of 500/s. When [E]t = 40 nM and [X] = 20 uM, the reaction velocity is 12 uM/s. Calculate Km.

Find Vmax first: Kcat=Vmax/Et

Vmax = Kcat(Et)

Vmax = 500/s(0.04 uM) = 20 uM/s

12 uM/s = [20 uM/s x 20 uM]/Km + 20 uM

Km = 13.3 uM

6. An enzyme catalyzes the reaction A ↔ B. The enzyme is present at a concentration of 4 nM, and the Vmax is 28 mM/s. The K_m for substrate A is 8 mM. Calculate K_{cat}.

Kcat = Vmax/Et

Kcat = 28 uM/s/.004 uM = 7000/s

7. A research group at CB3 (under the stadium) discovers the reaction Gobigred ↔ championship by Huskerase, a special enzyme required for winning football games. They do the following to characterize the enzyme. They find that V_{max} is 5.2 mM/s with [E_t] at 2.9 nM. What is K_{cat}?

Kcat = Vmax/Et

5200 nM/s/2.9 nM = 1793/s

8. In another experiment, with [E]t at 2 nM and Gobigred at 45 uM, they find that Vo is 600 nM/s. What is the Km of Huskerase? Hint: Since [E]t has changed, you will need to find the new Vmax.

Kcat = Vmax/Et

Vmax = Kcat(Et) = 1793/s (2 nM)

Vmax = 3.6 uM/s

0.6 uM/s = 3.6 uM/s (45 uM)/(Km + 45 uM)

Divide both sides by 3.6 uM/s, cross multiply and solve for Km

0.6/3.6 = 45 uM/(Km + 45 uM)

Km = 225 uM

1. Gensing is a competitive inhibitor for Type 9 oxidases. The K_I for 20 uM Gensing is 48 uM. What is the true Km of Type 9 oxidases with its natural substrate when the Km-app is 100 uM?

Figure out alpha first.

□=1+I/Ki……□= 1+20/46 = 1.42

Since □=Km-app/Km then Km=Km-app/□ then 100 uM/1.42 = 70.4 uM

2. Sooner is a competitive inhibitor for Huskerase. It is very difficult to purify Huskerase without Sooner contaminating the preparation. The K_I for 7 □M Sooner is 14 □M. What is the true K_m of Huskerase with its natural substrate, Gobigred, when the K_m^{app} is 56 □M?

Alpha = 1 + [I]/KI alpha = 1 + 7 uM/14 uM = 1.5

Km-app = alphaKm 56 uM = 1.5Km = 37 uM

3. Refer to the graph

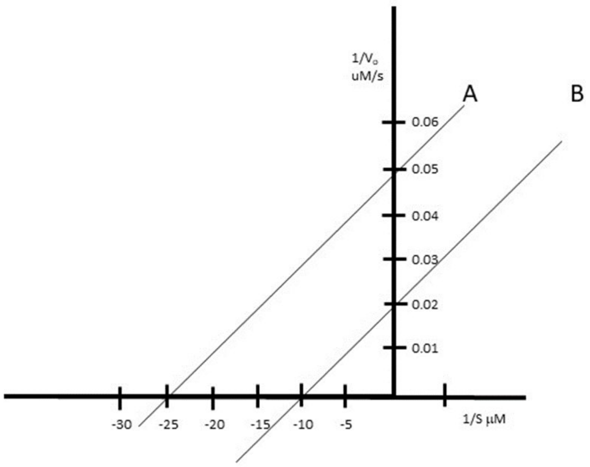

i. Which of these lines likely represents the inhibitor?

A

ii. What type of inhibition is this?

Uncompetitive inhibition

iii. What is Vmax with inhibition?

20 uM/s

iv. What is Vmax without inhibition?

50 uM/s

v. What is Km with inhibition?

0.04 uM

vi. What is Km without inhibition?

0.1 uM

The following table indicates the rates at which a substrate reacts as catalyzed by an enzyme that follows the Michaelis-Menten mechanism (1) absence of inhibitor; (2) and

(3) presence of inhibitor at 10 mM concentration of each of two inhibitors. Assume [E]t is the same for all reactions.

[S] (mM)	(1) Vo uM/s	(2) Vo uM/s	(3) Vo uM/s	1/[S] (mM)	(1) 1/Vo uM/s	(2) 1/Vo uM/s	(3) 1/Vo uM/s
1	2.5	1.17	0.77	1	0.4	0.85	1.3
2	4	2.1	1.25	0.5	0.25	0.48	0.8
5	6.3	4	2	0.2	0.16	0.25	0.5
10	7.6	5.7	2.5	0.1	0.13	0.18	0.4
20	9	7.2	2.86	0.05	0.11	0.14	0.35

Determine Km and Vmax for the enzyme with and without inhibitors.
Determine the type of inhibition.
Determine the Ki or K'i.
First do the 1/[S] and 1/Vo values for each column
Next, plot 1/[S] on x-axis and 1/Vo on y-axis
No inhibition: Km=3.3 mM and Vmax is 10 uM/s
For inhibitor 2 (competitive) the Km is also Km-app which is about 7.7 mM and Vmax-app is 10 uM/s
So Km-app = aKm which is 7.7 mM = a 3.3 mM and a = 2.3
Since a = 1 + [I]/Ki this would be 2.3 = 1 + 10 mM/Ki and Ki equals 7.7 mM.

For inhibitor (3) (noncompetitive/mixed) the formula to find a is 1/Vmax-app = a'/Vmax so a'=Vmax/Vmax-app which is 10 uM/3.3 uM which is 3.
So a'=1+[I}/K'I so this is 3=1+10/K'I and K'I is 5 mM.
